# Experiences of Using Online Peer Forums Among People With Postpartum Psychosis: Interpretative Phenomenological Study

**DOI:** 10.2196/80717

**Published:** 2025-12-24

**Authors:** Katherine Williams, Fiona Lobban, Elizabeth Chamberlain

**Affiliations:** 1 Department of Health Research Doctorate in Clinical Psychology Lancaster University Lancaster United Kingdom; 2 Division of Health Research, School of Health and Medicine Spectrum Centre for Mental Health Research Lancaster University Lancaster United Kingdom; 3 Perinatal Mental Health Team Liverpool and Sefton Perinatal Mental Health Service Mersey Care NHS Trust Liverpool, England United Kingdom

**Keywords:** postpartum psychosis, puerperal psychosis, postnatal psychosis, online peer support forums, online forums, peer-to-peer support, peer support

## Abstract

**Background:**

Although research has found online peer support forums to be helpful for those with mental health conditions, no studies have explored the experiences of those who use forums for support with postpartum psychosis (PP) specifically.

**Objective:**

This study aimed to understand the lived experiences of using online forums for PP, and how this form of support differs from professional and other informal support.

**Methods:**

This study used a qualitative approach, including semistructured interviews with 8 participants. Recruitment took place via an online forum run by a charity called Action on Postpartum Psychosis. Transcripts were analyzed using interpretative phenomenological analysis.

**Results:**

Four themes were developed in line with participants’ experiences (1) from isolation to connection: validation, growth, and hope from shared experiences; (2) complementing not replacing: filling the gaps in support; (3) impacts of privacy, representation, and readiness to share on engagement; and (4) relational experiences within peer support: altruism, boundaries, and comparison. All participants believed forums were helpful to their well-being and recovery; however, some also reported difficulties with engagement, comparison, and regulating their own use. Findings suggest that forums may benefit from being designed in a way that protects users and their identities, for example, via trigger warnings and setting boundaries.

**Conclusions:**

Peer online forums offer a unique and potentially effective addition to existing support provided by professionals and personal connections. Professionals should signpost people experiencing PP to forums, but should also understand the support that may be needed in terms of monitoring use and ensuring that appropriate boundaries are put into place.

## Introduction

Postpartum psychosis (PP) is a rare perinatal mental health condition, affecting between 1 and 2 in 1000 individuals [1,2]. It is a psychiatric emergency that can occur suddenly [3], usually between 0 to 185 days following childbirth [4]. Symptoms characterizing PP include mood changes, hallucinations, and delusions [5,6]. The risk of onset of psychosis is thought to be approximately 23 times higher in the 4 weeks following childbirth than at any other stage of life [7]. PP is less common and less understood than more common perinatal mental health conditions, such as postnatal depression, and may therefore be less readily recognized [8]. Current understanding of what is helpful for PP is limited [9], and therefore it is important to consider support for people with lived experience of PP throughout and following this period.

Despite recent introductions of specialist perinatal support within the United Kingdom [10,11], and the time spans for which these services can support individuals being extended [12], gaps remain in both understanding and treating PP [4,13]. People with lived experience of PP have reported a lack of awareness from both the public and professionals [14], leading to delayed recognition and treatment [15]. PP is not formally recognized as a diagnosis by the National Institute for Health and Care Excellence [16] or the *DSM-5 (Diagnostic and Statistical Manual of Mental Disorders* [Fifth Edition]) [17]. The most common treatment options for PP include medication [18], psychological interventions [19], and, occasionally, electroconvulsive therapy [20]. Recovery from PP is thought to take between 6 and 12 months [21], with the most severe symptoms often resolving within 12 weeks when intervention is received [22]. However, there are many challenges that people with lived experience of PP encounter that these traditional interventions may not address. People with lived experience of PP report feeling isolated [14], overwhelmed [5], and stigmatized [19,23], indicating that psychosocial challenges for people with lived experience of PP are difficult to navigate throughout the recovery process.

An increasingly popular means of support for health-related challenges is through online mediums, and the use of this for mental health has become a fast-growing area [24]. It is thought that online support could be particularly helpful for mental health for reasons including the opportunity to remain anonymous when seeking support [25] to help decrease felt stigma [26], and the social isolation that is also commonly experienced [27].

Peer forums are a form of online support, characterized by the ability to anonymously provide and receive support among those in similar situations [28]. Previous research has found that peer support forums can reduce isolation [29,30], promote information sharing [30,31], and provide emotional support and hope [32]. In a recent realist synthesis to identify the impacts of online peer support forums for mental health [29], findings included improving self-efficacy and psychological safety, and connection. However, users of online support have also reported that they can sometimes feel triggered by others [30], receive misleading, defensive, or unempathetic responses [31], and struggle with their own boundaries [30], suggesting this may be a nuanced area.

When considering online support for those with PP, it is important to consider the unique experiences that could impact its use. Declines in mental well-being within the perinatal period can be distressing and overwhelming [33]. In addition, there is the challenge of navigating parenthood and the changes in identity that this and PP bring [19], alongside the impacts of symptoms on daily life, with those who experience relapse of PP being found to have more ongoing functional difficulties [34]. Online forums for support with parenting are commonly used for voicing feelings around parenting that might feel difficult to speak about openly in other contexts; however, research has found that forums tolerate these expressed emotions only to a certain level before it is implied the person may not be in the “norm” [35]. This limits the support available to people with lived experience of PP in these nonspecialist forums and suggests they might need to speak with others who understand to feel safe.

Despite there being no existing research that specifically examines the impact of peer online support for people with lived experience of PP, studies on wider topics have reported related findings. For instance, perceived social stigma is correlated with increased use of online support [36], and those experiencing perinatal mental health conditions tend to have higher levels of internal stigma [33]. Furthermore, findings have reported that increased use of forums could result in users being more likely to disclose their symptoms to professionals [33]. Research has demonstrated that those who conversed with others who have also experienced PP found this support helpful and normalizing [19]; however, with PP being rare, shared experiences may likely be more easily accessible through online mediums. Stawarz et al [37] suggested that technology and online support should not be a replacement for more formal support but instead should complement it and form part of a wider, holistic system. This may be particularly true for PP, which requires fast intervention and support [38], indicating the need for professional involvement alongside personal and social support.

NHS guidance directs people with lived experience of PP to peer support forums [2]; however, there have been no studies that have explored the impacts and safety of this type of support. Therefore, this study aims to explore the experiences of those who have used online peer forums for support with PP, to increase the evidence base and aid professionals in signposting decisions. For this research, support with PP was defined as when an individual had used the forum specifically in relation to any symptoms or difficulties related to their PP. The research questions posed were: (1) How do individuals with lived experience of PP use and make sense of online peer forums? and (2) In what way does online support differ from support from health care professionals and other informal support?

## Methods

### Study Design

In this study, Interpretative Phenomenological Analysis (IPA [39]) was used to explore the lived experiences of using online forums for PP, and how this form of support differs from that of professional and other informal support.

This included any support received within a forum environment that was related to symptoms or difficulties associated with PP. This broad definition of support meant that participants were able to speak about their own individual support needs related to their experiences of PP, which can vary between participants. In this study, “support” was operationalized as any form of emotional, informational, or experiential connection participants received through engagement with an online peer forum.

The method used for this study was IPA. This method most appropriately met the research aim to generate a rich and detailed understanding of participants’ experiences, rather than a broader and more varied focus, as is seen in other qualitative methodologies such as thematic analysis [40]. In addition, within IPA, there is a focus on lived experience, which helps to capture nuance between participants [41] and to explore the meaning behind experiences [42].

### Patient and Public Involvement

Patient and public involvement (PPI) has been found to add insight from lived experience, achieve meaningful outcomes [43], make research more accessible, and ensure sensitivity to the topic [44]. The lead researcher held consultation meetings with a member of the public who had lived experience of using forums for support with PP. Consultations included input on the topic guide, completing a mock interview, and developing research materials. In addition, the researcher liaised with employees of Action on Postpartum Psychosis (APP) with lived experience of PP regarding research materials, the topic guide, and terminology used. In response to PPI feedback, wording in the research materials was changed, alongside edits to the research flyer to ensure language was accessible.

### Inclusion and Exclusion Criteria

Inclusion and exclusion criteria were considered to ensure that the sample was relevant and focused on the research aims (Table 1).

**Table 1:** Inclusion and exclusion criteria.

Inclusion or exclusion	Criteria	Rationale
Inclusion	Aged 18 or older Having the capacity to consent Having used peer support forums within the past 2 years Symptoms of postpartum psychosis (PP) experienced at any time	Able to give informed consent Ensuring consent is ethically given Forums are evolving quickly [45]; those who used forums longer ago may have had experiences less relevant to current experiences Due to recognition that people with PP can need support for several years after onset, and recognizing that support has changed in recent years [11,12], and wanting to capture information from those who used the forums when support might not have been as available
Exclusion	None beyond self-reported experiences of PP symptoms	No formal mental health diagnosis required as PP is not recognized as a distinct diagnosis [17], and potentially issues of stigma [46] and lack of formal screening measures leading to PP going unrecognized [20]

### Sampling

A purposive sampling approach was used between February and July 2024 to recruit people with lived experience of PP who had used forums for support. This method aligned with the aims of IPA, which prioritizes depth of experience over breadth of participants to gain a detailed understanding of the phenomenon of interest. A sample size of between 8 and 10 participants was sought, in line with recommendations for both qualitative research [47] and research conducted at a doctoral level [36]. It was anticipated that this would provide sufficient data to build an understanding of experiences of support, particularly due to the rarity of PP and therefore the likely small cohort of available potential participants who had both experienced PP and used forums for support with this. Furthermore, this sample size was considered sufficient to answer the research questions posed.

### Recruitment

Participants were recruited through an international forum run by APP. This forum is hosted by HealthUnlocked, a worldwide community providing support for over 250 health conditions, with the APP forum accommodating 3793 users (figure correct on February 19, 2025). Participants could have accessed other forums for support with PP and still participated, as the research was not specific to the APP forum. Meetings were held with moderators of the APP forum, who posted a recruitment flyer on the forum to advertise the study. Forum users interested in taking part were invited to contact the lead researcher via email and then offered a screening call to check eligibility and to address any queries.

### Study Procedure

Following the initial screening call, eligible participants were provided with an information sheet (Multimedia Appendix 1) and a consent form via email (Multimedia Appendix 2). Participants were then asked to return the consent form to the lead researcher if they wished to take part. Once the form was returned, an interview time and date were booked that were mutually convenient for both parties. Sociodemographic information (Table 2) was collected verbally at the start of each interview, and participants were given the choice to decline to answer any questions if they wished.

**Table 2:** Participant demographic information.

Participant	Pseudonym	Age (years)	Ethnicity	Country of residence	Time since onset
1	Jane	31	White Scottish	Scotland	2 years
2	Florence	39	White British	England	13 months
3	Emmeline	57	White American	United States	22 years
4	Frida	34	White British	England	2 years and 3 months
5	Ada	35	White, Mixed Asian	England	4 years
8	Rosalind	51	Black African	England	19 years
9	Marie	35	African American	United States	1 year and 7 months
10	Eleanor	43	White British	England	8 years

A topic guide (Multimedia Appendix 3) was used during the interviews to remain close to the research aims. The definition of support with PP was used to develop this topic guide, and the questions were reviewed and refined within research supervision and PPI meetings. A mock interview was conducted with a PPI representative, which led to some changes in the terminology and style of the questions within the topic guide, for example, reducing jargon in the questions.

### Data Collection

Interviews took place at an agreed time over Microsoft Teams, Zoom (Zoom Communications), or a telephone call, depending on the participant’s preference, and were recorded onto an encrypted audio device. Before commencing the interview, consent was checked. A topic guide was used as a prompt; however, in line with IPA [39] methodology, the interviews were guided by participants and their experiences [48]. This was done by allowing participants to speak about the phenomena they felt were pertinent to their experiences of using forums for support with PP. Participants were able to interpret questions in their own way, and further questions followed from responses given, meaning that a more thorough exploration of experiences could be undertaken. This helped to gain further context and understanding around the topics discussed by each participant, as well as a way to relate any responses given back to the research questions. Following the interview, participants were debriefed, and their safety was checked.

### Data Analysis

To analyze the data, the first 3 interviews were transcribed by the lead researcher soon after completion to review the questioning style. The remaining 5 interviews were transcribed by a contracted transcriber, who had undergone screening and signed a confidentiality agreement. The reason for this was that the lead researcher was not feeling immersed in the data by transcribing, but instead by reading the transcript back while listening to the recording. As a transcriber was available, this was used to spend more time engaging with each interview. Reading the transcripts while listening to the interviews helped to understand context and emotive language and to become more familiar with the data, while also checking the accuracy of the transcripts. Analysis was performed in line with recommended processes from Murray and Wilde [48], which were developed for postgraduate IPA projects undertaken by those who are novices to IPA. This approach follows a stage process: first, initial coding, which involves notations for any elements directly relevant to the research aims. This process was completed by hand using Post-it notes. The second stage is grouping the codes into subthemes, with each subtheme given an interpretative summary and title. The following stage is producing interpretative summaries and titling themes for each participant. Following this, the stage of merging analyses across transcripts was completed, where the subthemes from each participant were merged into collective themes across participants, with narratives and titles created for each.

### Reflexivity

IPA is a methodology underpinned by a critical realist epistemology [41]. This aligned with the lead researcher’s critical realist view that, while the material world exists, our own perceptions and language shape the way in which we see and experience it [49]. This means that experiences of the world are all individual, such that although all participants had experience of using online forums, the meanings made from this would all differ.

The nature of IPA involves a process of double hermeneutics, with the participants interpreting their own experiences and the lead researcher further interpreting this themselves [49]. Therefore, the data output from this study is a secondhand account of the data collected, which may have been influenced by the demographics of the researchers (Multimedia Appendix 4). The lead researcher was a White, UK-based female trainee Clinical Psychologist with a professional background in social work. These personal and professional identities shaped the lens of focus when approaching the research, potentially influencing the analysis by orienting attention toward psychosocial, relational, and systemic elements of the data. In addition, the analysis was conducted from within a Western sociocultural context, with UK-specific mental health systems and norms likely influencing interpretation. While the researcher had no personal experience of PP, previous clinical work with individuals experiencing PP may have led to certain preconceptions or expectations.

To try to minimize the impact of these factors and enhance the trustworthiness of the analysis, several measures were taken. First, regular supervision was sought throughout the research process. Two supervisors supported the project; both were qualified clinical psychologists who were White, female, and living within the United Kingdom. One supervisor had expertise in qualitative research methods, while the other had experience working within perinatal mental health services. Within supervision, they both reviewed developing codes and themes and offered critical feedback to help challenge assumptions and ensure that interpretations were grounded in participants' accounts rather than shaped by the researcher’s preexisting frameworks.

The lead researcher also engaged in reflexivity through the use of a reflective diary, in which decision-making, emotional responses, and interpretations of the data were documented. This process helped identify where professional training or clinical experience might be influencing the interpretative process. Reflexivity was particularly prioritized after emotionally resonant interviews or when strong thematic impressions emerged.

In addition, moments of purposeful reflection were built into the analytic process—for example, pausing after the first interview and again after the first full transcript analysis, supported by supervision. This helped to preserve the integrity and transparency of the analysis and to ensure that findings remained grounded in participants’ lived experiences.

### Ethical Considerations

Ethical approval was granted by the Lancaster University Ethics Committee (FHM-2024-3722-RECR-3). An information sheet was provided to participants, and informed consent was obtained through a signed form. Participants were given the option to opt out at any point before data analysis was completed. Due to the potential for distress associated with the interview process, debrief materials were sent to all participants following the interview, with details of services they could contact for support if necessary. Contact details for a named professional involved in each participant’s care were collected before the interview in case any risk issues were disclosed during the interview process. Data were deidentified and securely stored on the university’s cloud storage. Participants were compensated with a £30 ($39.95) payment for giving their time to the research.

## Results

### Participant Characteristics

Initially, 15 people expressed interest in the study. Of these, 2 did not attend the initial screening call, and 3 were ineligible to participate due to not using forums for PP (n=2) or not using forums within the past 2 years (n=1). Two participants consented to be interviewed; however, they did not take part due to disengagement (n=1) and withdrawal (n=1). This resulted in a final number of 8 participants. Recruitment was a relatively easy process, with enough people showing interest through the APP without the research having to be advertised elsewhere. Participants were happy to discuss their experiences openly, with the average length of interview being 41 minutes. Participants were given pseudonyms, and demographic information was collected (refer to Table 2).

### Analysis

Following the analysis, 6 interrelated themes were constructed to illustrate experiences of using a peer support forum. Multimedia Appendix 5 demonstrates which participants contributed to each theme.

### Theme 1: From Isolation to Connection: Validation, Growth, and Hope From Shared Experiences

All participants are represented within this theme, which captured the evolving relationship participants had due to PP; beginning with feelings of isolation and stigma, progressing through validation and understanding when finding the forum, and resulting in a sense of growth, knowledge, and purpose. Validation of these experiences from peers who had shared similar challenges was identified as an important part of recovery.

“Reading the experiences of women who’d recovered gave me hope that I would too”

Florence

“I think it’s given me a real validation that what I went through was real… and not just something they couldn’t figure out”

Emmeline

This demonstrates the lasting impact of feeling misunderstood during the initial phases of PP, and how finding the forum, even many years after experiencing PP, helped to provide affirmation. For participants who experienced PP longer ago, the impact of having many years of no connection to other people with lived experience of PP was loneliness that was turned around by finding the forum.

The accessibility and anonymity of the forum made it particularly valuable. It allowed participants to engage when it felt emotionally safe and to return to the space whenever needed without pressure or stigma.

“I think the other thing is the accessibility day or night, in that if… maybe I couldn’t sleep or I was up with the baby, you can access it at any time.”

Florence

While many participants used the forum from the early days of diagnosis, Marie did not use the forum initially due to feeling in denial. She described how the forum helped her to accept her diagnosis, leading to her eventually being able to post herself.

“You’re assisting people… whilst still receiving treatment for yourself, so yeah, I felt so strong again in the community”

Marie

The rarity of PP also impacted experiences, with participants feeling as if there was no one they could speak to before the forum who understood what they had been through.

“That’s a long time to go without ever having talked to somebody about something that was so pivotal in your life, and so that was just amazing to me when I first got on and started talking to people. The feeling of validation, the feeling of connection, the feeling of wanting to help was overwhelming.”

Emmeline

The use of the word “pivotal” here demonstrates the profound impact that forums can have for those who use them.

Two participants commented on how they had felt increased isolation due to others cutting them off following their experiences of PP:

“I’ve been shunned by people who I thought were friends”

Rosalind

“I felt isolated from everything, from the community, from my family”

Marie

Rosalind and Marie were also the only participants in the study with African heritage, which suggests it is worth considering the specific cultural impacts that PP can have and how PP might be viewed differently within different communities. For these participants, the isolation was felt on an additional level due to personal support feeling unavailable, highlighting how important the connection on the forum was.

### Theme 2: Complementing, Not Replacing: Filling the Gaps in Support

All participants, except one, are represented in this theme, which recognizes the way in which forums supplement existing services and personal support. Peer support was experienced differently from any other support that had been offered, much of which had led to people with lived experience of PP feeling misunderstood and judged.

“[Peer support] is almost like a superpower because… you’ve got that empathy”

Frida

Even for participants who felt there had been a good support system around them throughout, it felt safer to speak to peers, with the benefit of feeling less like a burden:

“You don’t want to burden your family either, so talking to your people on an online forum… is, I think, personally the better way.”

Eleanor

There was a sense of safety felt within the PP community when speaking about these distressing thoughts, which may not be present elsewhere.

Personal support was felt to be lacking in the understanding that peer support can provide:

“I’ve always been so lucky to have friends and close family and support… but for this experience, it’s not been good enough.”

Jane

This demonstrates how there is a unique nature to PP that means personal support might not have the same impact when experiencing severe mental health difficulties. Jane describing how “*everyone close to me has had a different kind of experience of the same situation”* shows how PP adds a layer to the changes felt post partum.

When referring to professional support offered, most participants found this helpful and felt that such services should be complemented by the forums rather than replaced. There were concerns raised about people who might use the forums instead of speaking to professionals.

“Some of the women sounded like they just really needed to go and see their doctor, and that this forum wasn’t necessarily quite the right place for them.”

Jane

This highlights how PP is multifaceted and that one type of support may not be helpful in isolation. There is recognition of the limitations that the forum can have, particularly around medication and interventions. The acknowledgment of health care professionals being central to the start of recovery demonstrates how forums may become more useful at a later stage.

However, not all participants felt supported by professional services. Rosalind described how she felt let down by services, stating that she felt misunderstood. Rosalind believed that services are so overstretched that they cannot provide the support needed:

“It’s ridiculous that we’re having to use peer support in order to provide services that really the NHS should be providing. It’s the walking wounded trying to help each other.”

Rosalind

Rosalind’s experiences may be different from those of other participants due to witnessing changes in services without having access to more specialist services that have recently been created (eg, being ineligible for MBU support or perinatal services support due to experiencing PP 19 years ago). As someone still experiencing the impacts of PP, Rosalind demonstrates a need for longer-term support than statutory services can provide. This was reinforced by Jane describing discharge from professional services as being a *“carpet from underneath you.”* It was felt that forums could help to fill this gap, as they can be accessed for as long as needed:

“Then once I get discharged from that, I’m like, ‘right, I’ve still got my platform, I’ve still got the online stuff that I can still reach out to should I need it’… it’s like you feel safe… you’re not left to it.”

Ada

This shows a potential disconnect between what specialist services provide (up to 24 months post partum) and what people with lived experience of PP believe would be helpful.

### Theme 3: Impacts of Privacy, Representation, and Readiness to Share on Engagement

Two participants are not represented in this theme, which recognizes that although forums were valued by all participants, state of mind, age, and identity all played a role in how able people felt to share their experiences openly. Reflections were provided on the stages at which participants first started to use the forums, with Ada referring to how, initially, she felt paranoid and embarrassed and therefore did not want to use them. Eleanor echoed this point, stating that she did not feel able to speak about PP until 3 months following the onset. There were physical elements of PP that were thought to be involved in engagement, alongside being able to check in on emotional state.

“Then at the time when I was so acutely unwell, my brain was just firing off left and right, I could barely even use my phone at one point, and I couldn’t organize my thoughts.”

Eleanor

This adds weight to the importance of the type of support at different stages, acknowledging that symptoms of PP can make using forums more difficult and therefore other support may be imperative at this time. Privacy within forums was also a challenge, with the anonymity often provided not removing this concern:

“[Forum] is an international forum, and I do worry a little bit about privacy issues.”

Emmeline

Florence referred to younger generations potentially finding forums easier to use, but in older generations, it is more common to keep one’s feelings to oneself. As the interview progressed, Florence reflected on this and provided insights into how she can often still feel shame around PP, wondering if it could be helpful for her to open up more. This demonstrates the complexities of deciding what to share within forums and how this might continually shift as participants move through their recovery.

A nuance that came through within this theme was around ethnicity, with Rosalind feeling that there are impacts of being a Black woman on her use of forums. Rosalind described how Black women are more likely to experience birth trauma and be within the mental health system, and how this could translate to being more likely to use forums for support. However, this was not reflected in Rosalind’s experience, with feelings of being underrepresented within the forums, which resulted in further isolation.

“If [forum] could have someone who is black, that would really help me as well, because I think in the black community, mental health isn’t talked about or understood.”

Rosalind

This demonstrates how identity can further lead to feelings of isolation and how this can replicate difficult experiences from other areas of life. In feeling unable to relate to others on the forum, there could be an impact on engagement and freedom to share, causing those from a global majority background to be at a disadvantage when engaging in peer support.

### Theme 4: Relational Experiences Within Peer Support: Altruism, Boundaries, and Comparisons

One participant is not represented within this theme, which acknowledges the complexities that navigating a community can bring. On one hand, users could access information and support, and were given opportunities to use their experiences to help others. However, sometimes these opportunities brought pressure to respond and a sense of responsibility or comparison to others.

The forum was described by Marie as a “*happy place*” that allows participants to get their own support as well as give it:

“I just want to reach out… and tell them that they can get through it, there is an end, and I am proof there is an end.”

Emmeline

This acknowledges the tie people with lived experience of PP tend to feel to the community, wanting to give back and *“do my bit for the community”* [Frida]. However, it also felt at times that this desire to give back became burdensome:

“The compulsion to post or to reply to people… that did cause me quite a bit of stress. Those feelings of like guilt.”

Frida

The use of the words *“compulsion”* [Frida] and *“obsession”* [Florence] were used when speaking about using the forums when in a more vulnerable place. This shows how forums can have emotive impacts on those using them, who may already be experiencing their own distress and challenges. There was mention of moderation at these times being helpful:

“He’d [partner] realized what had been going on with me, going down a rabbit hole on the internet, obsessively looking for answers… so there was external moderation.”

Eleanor

Boundaries were also described to help safeguard against feelings of responsibility. However, they were also acknowledged to be difficult to establish while mood is fluctuating, emphasizing the impact that symptoms of PP can have on engagement.

A further challenge of the forums arose from the tendency to compare. Upwards comparison was the most prominent in interviews, characterized by feelings of being patronized or not being good enough when speaking to those who had an *“easy”* [Jane] recovery.

“You think have I made a mistake? It’s like am I doing it wrong? Like am I doing recovery wrong?

Jane

Ethnicity also played a role in upward comparison, demonstrating the impact that diversity within spaces such as forums can have.

“I just think in white women the care is generally a lot better, and I’m seeing women recover so much better, whereas with me now I’m disabled and it’s ruined my full life.”

Rosalind

This shows how forums can highlight discrepancies in care offered for PP and recognizes the impact that this could have on users.

Downwards comparison was also mentioned during interviews and seemed to be associated with feelings of being ungrateful for their experiences, feeling sad for others, and wondering “what if.”

“I feel fortunate with the care that I got… but sometimes it makes me feel… ungrateful.”

Jane

Questions arose about how representative forum posts are of the wider population of people with lived experience of PP. For example, Jane wondered if those who are struggling more tend to use forums more, interpreting this as feeling that those who recover well move on.

“It’s just scary to see so much content and relapses and stuff, but that might be the people who might need support. I wonder if there’s like a hoard of people who have not had like relapses or other diagnoses that don’t need the support and are fine and not on the forum.”

Jane

This poses an interesting point about the types of users that forums might attract, and whether this is reflective of different experiences of PP, or whether this tends to reflect certain narratives instead, such as people struggling to recover. However, a differing opinion was put forward by Rosalind, who felt as if there were not many people posting who were reporting difficulties, which left her feeling as if she was struggling more than others:

“At least online it feels like they have recovered more than I have… that’s why I wish people would talk because I can’t be the only one who’s had an adverse reaction to their meds.”

Rosalind

## Discussion

### Principal Findings

This research aimed to explore the lived experiences of using online forums for PP and how this form of support differs from that of professional and other informal support.

The findings demonstrate that this is a complex and nuanced area, and that people with lived experience of PP tend to use forums when reaching stability rather than in the initial acute phases of PP. It is also acknowledged that the use of forums can be helpful, supportive, and aid recovery. However, forums can also have challenges such as feeling responsible for other people with lived experience of PP, comparing to others, and needing to implement boundaries to maintain well-being. Individual experiences are impacted by many factors, including identity, other available support, and recovery. The research was novel in that it was the first investigation into the use of peer online forums for PP, adding to the limited research base on PP. The use of IPA allowed an in-depth, idiographic insight into the lived experiences, which did not just focus on forum use at a single point in time, but instead over the recovery trajectory of the participants. This created an understanding of how the use of forums changes, increasing knowledge of how these mediums can provide longer-term support for people with lived experience of PP.

An important element of this research was related to the rarity of PP [6], which was felt to increase the isolation that can be felt within perinatal mental health conditions [50]. Online forums give the opportunity for connection between people with lived experience of PP that otherwise may not occur, especially if situated in a more remote area. Connection found within forums was discussed by all participants, which demonstrates the extent to which online relationships can be felt as true connections despite potentially never meeting face-to-face. A recent systematic review has found PP to be a relational experience, both in recovery and management [51], and although this was regarding the couple relationship, considerations should be given to how the relational elements of PP might also be helped by connection on forums and with peers. The ability to engage on forums was found to be helped by accessibility and anonymity; this mirrors existing findings regarding forums that these factors remove barriers to support and reduce stigma [30]. This is particularly important within the PP community due to increased levels of stigma [52] and fears around social services involvement following disclosures [53].

Connection was felt to be an imperative part of forum use, linking to the CHIME recovery framework [54]. Forums played a clear role in promoting connectedness, instilling hope, shaping identity, and offering a platform for meaning-making and empowerment. However, it is important to note that this was related to personal recovery and that there are differences between this and clinical recovery [55], with both being important within psychosis presentations [56]. This contextualizes theme 2 and the importance of both professional supports to promote clinical recovery, while peer support can help achieve personal recovery.

However, connection can come with challenges, such as potentially feeling responsible for the well-being of other users. A key finding within this study was that participants felt they wanted to give back to the PP community, which was highlighted by several participants as being important for both their recovery and well-being. Although previous research has referenced helping others as potentially being related to reciprocity, and therefore expecting mutual benefit from this [57], within this study, participants suggested different reasons for this altruism. Particularly, this stemmed from an understanding of “dark” times and wanting to provide hope when approaching recovery. For participants who still felt in the midst of PP, helping others originated from a place of not wanting anyone else to feel the isolation and loneliness they experienced. Despite feeling that helping others was important to them, it is crucial to recognize the impact that this responsibility could have on forum users.

The results reflected how participants felt it was important to have boundaries when using online forums, and how this had to be learnt. This was found to be crucial during acute stages [30], which is particularly relevant as participants often engage on forums shortly after the onset of PP. Within the data collected, participants acknowledged how boundaries were more difficult to implement when experiencing more severe symptoms of PP. This poses a question of how forum users set boundaries during the time they potentially need them the most. Participants described feeling unable to step back, which resulted in obsessively scrolling for hours. This is similar to current understandings of social media addiction [58], with peer online forums being alike in characteristics; involving connection, community, and gaining information [29]. Understandably, this sensation akin to addiction could be felt more severely by people with lived experience of PP, who are potentially experiencing a lack of connection due to sleep deprivation [59] and symptoms of mania [7].

Conversations regarding professional support took place across countries and cultures, where participants explained how forums were helpful to them because they could access them for as long as was required; in turn, this helped ease the anxiety of being discharged from professional care. Research has found that levels of anxiety are often high at discharge from both hospital and community care [60,61], which could be particularly relevant within the United Kingdom, where specialist perinatal support is only provided for the first 2 years following birth [12], despite symptoms and impacts of PP sometimes continuing well past this point [62]. This links to Jane’s feelings that the forum has an overrepresentation of people with lived experience of PP who have found recovery more challenging, as these users may have required more support and have therefore continued to use the forum. However, a related study recently reported that they did not find engagement with online peer support forums to be correlated with symptoms or disability [63], which makes it important to consider why people with lived experience of PP might continue to use forums.

The findings of the study showed that many of the participants felt they engaged in comparisons with other users on the forum. This can be understood within the context of social comparison theory [64], which has been found to have a strong presence in the use of online communities, such as social media [65]. While upwards comparison has been previously linked with promoting hope and downwards comparison has been thought to put things into perspective [66], this study found comparison to also have potentially detrimental effects. Upwards comparison resulted in the perception of insufficient recovery, while downwards comparison resulted in feelings of guilt about being ungrateful, as their situation could have been worse. It is important to understand the impact that both types of comparison may have on users, particularly if using the forum at a more vulnerable point.

### Strengths and Limitations

The use of IPA as a methodology was found to be of value within this study, allowing the understanding of not only the commonalities between participants’ lived experiences, but also divergences from these and an exploration of why these might occur. In addition, even with a small sample size, there was diversity in the ethnicity of participants, which contributed to understanding how culture may impact experiences. Using PPI within the research was also felt to be a strength, as this added the perspective and input of people with lived experience of PP, which allowed adjustments to the research when needed. The study added to the lack of existing research around PP to help increase awareness, understanding, and intervention options for people with lived experience of PP.

This research had some limitations in terms of its design. Participants in the study were recruited via one peer support forum for PP, which, although international, was based in the United Kingdom. Due to the recruitment methods, likely, the people who saw the flyer were still using forums, which indicates that they are more likely to have found the forum helpful or that they still needed support. Therefore, there may be a demographic of people who are not represented in this research. Although recruitment was only through one forum, participants were able to speak about experiences from any forums they had used for support with PP; however, this was not recorded. This meant that it is difficult to ascertain whether particular forums are more helpful than others or what characteristics of forums are more useful. In addition, all participants were aged between 31 and 53 years old, and an additional point made by Florence—that younger generations tend to find it easier to share online—poses questions around whether there are age differences related to how people use forums for support.

Despite recruitment taking place on an international forum, participants all resided in Western societies. Perinatal support within these countries can differ greatly from what is offered elsewhere [52]; therefore, it is important to acknowledge that these findings may not be generalizable beyond this population. In addition, it has been recognized and evidenced that those from the global majority can experience worse outcomes regarding mental health care [67], maternal support [68,69], and birthing experiences [70]. Within this research, there are views offered by 2 participants from a global majority background and one from a mixed ethnic background; however, this is a small proportion of the sample, and a more culturally balanced sample could have been beneficial to ensure a more comprehensive understanding of experiences.

Finally, the lead researcher of this study had not personally experienced PP. Within interviews, some participants described the value of shared experience, in that anyone who has not experienced PP cannot understand it. This highlights the impact of the researcher’s understanding and interpretation of the interviews as someone who has not shared these experiences. Consequently, the findings of this study may have looked different had they been analyzed by someone with lived experience.

### Clinical Implications

Following completion of the analysis, several recommendations were made related to the data.

For forums, trigger warnings and clear titles should be displayed on posts to ensure that readers are making an informed decision about what they are about to read. This links to theme 4, which acknowledged how safeguards against feelings of responsibility are important, as they help users to maintain appropriate boundaries and assess their own well-being before accessing content they might find upsetting.

A key consideration for forums is around representation and how this is managed within an online environment, as discussed within theme 4. Experiences related to diversity were central for the participants from the global majority in this research, with comments on how those within their families and communities did not understand, and how they felt shunned. There was also a reference to how, within the black community, mental health is not spoken about. This highlights a lack of spaces that people with lived experience of PP with black heritage can speak about their experiences. Given this, it might be helpful for forums to consider specific areas for the global majority to share their stories and experiences. This is supported by a recent study that found those from the global majority believed a space for peer support would help have open discussions and create a sense of belonging [71].

When considering implications for professionals, it would be helpful if professionals could signpost to forums where it would be beneficial for people with lived experience of PP. As noted in theme 2, forums have the potential to complement professional support; however, for people with lived experience of PP to use forums, they need to be aware of their existence. Signposting has the potential to reduce load on services, which was described by participants within theme 2 as an extra medium of support. However, it is also important that professionals recognize the potential for distress when using forums and help those they are working with to set boundaries and monitor usage. This is particularly important for people with lived experience of PP, who may be in the initial stages or who might be at risk of relapse.

Within future policies regarding perinatal mental health, it could be helpful to consider the expansion of peer support roles, helping people with lived experience of PP to guide while having services involved that help to safeguard their own well-being. Theme 1 recognized how people with lived experience of PP could go many years without connection with anyone who had also experienced PP and the impact this had on their feelings of isolation. Although PP peer support roles do exist, involvement of people with lived experience of PP is limited; increasing this support could help increase accessibility and extend it to those who may be less likely to use forums. In addition, this research has highlighted existing knowledge around perinatal services often being western-centric in their approach [72]. It would be helpful for future policy to consider how to increase cultural competence, to tailor support, and improve access for communities that may otherwise be underrepresented within services.

### Future Research

In the future, it would be helpful for research to more closely examine the longer-term impacts of PP. Participants within this study highlighted how specialist support was withdrawn at a time when help was still needed. Understanding the continued needs of people with lived experience of PP post recovery could help to improve service provision and increase appropriate signposting. In addition, future research related to the longer-term impacts of using peer online support forums could also be beneficial. As forums are a relatively new medium for support, the lasting impacts of their usage are unknown. As previously stated, the 2 participants within this study who had found the forum long after the onset of PP still found it beneficial. It could be helpful to understand why this might be, and how long people with lived experience of PP continues to engage with the forum, to understand what variables are involved in continued use.

Finally, with a limitation of this research being related to generalizability across cultures, future research should consider cultural differences when it comes to peer support for PP. It would be particularly useful to explore potential barriers to accessing peer support, representation issues, and how cultural perspectives of mental health play a role in disclosure and help-seeking.

### Conclusion

This research study demonstrates the impact that online peer forums can have for people with lived experience of PP , recognizing that they help to increase connection, provide unique support, and play a role in recovery. There are challenges associated with the use of forums, such as issues with representation, comparison with others, and a sense of responsibility for other users. Helpful considerations include forums introducing spaces for global majority users and including trigger warnings to help users maintain boundaries. In addition, it was suggested that increasing professionals’ awareness of forums as a channel of support and assisting people with lived experience of PP to access these would be beneficial to both services and service users.

## Appendix

Multimedia Appendix 1 Participant information sheet.

Multimedia Appendix 2 Consent form.

Multimedia Appendix 3 Interview schedule.

Multimedia Appendix 4 Example of double hermeneutics process.

Multimedia Appendix 5 Extract of individual theme for one participant.

Multimedia Appendix 6 Cross participant themes and quotes.

Multimedia Appendix 7 Table of summary of experiences of personal support and forum support.

## Abbreviations

APP: Action on Postpartum Psychosis
DSM-5: Diagnostic and Statistical Manual of Mental Disorders (Fifth Edition)
IPA: Interpretative Phenomenological Analysis
PP: postpartum psychosis
PPI: patient and public involvement
