# Supplementary material for: Experiences of Using Online Peer Forums Among People With Postpartum Psychosis: Interpretative Phenomenological Study
Source: JMIR Hum Factors. 2025 Dec 24;12:e80717. doi: 10.2196/80717 (PMC12780708; doi:10.2196/80717)
Supplement: Multimedia Appendix 1 [file humanfactors_v12i1e80717_app1.docx]

**
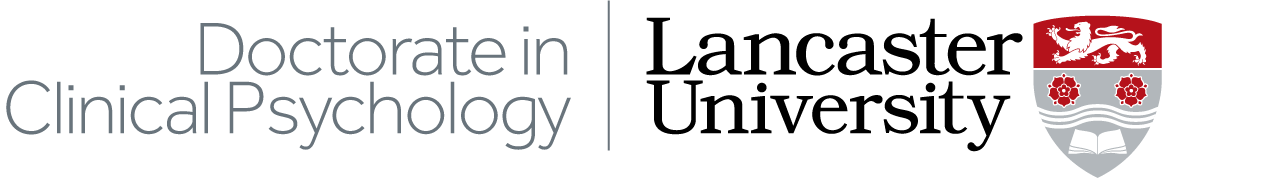
**

**Participant Information Sheet**

***Experiences of using online peer support for postpartum psychosis***

My name is Katherine Williams, and I am conducting this research as a Trainee Clinical Psychologist at Lancaster University.

**What is the study about?**

The purpose of this study is to learn more about the experiences that users of online peer support have had, specifically when using these resources/forums for support with postpartum psychosis.

**Why have I been chosen?**

You have been approached because the study requires information from people who have experienced symptoms of postpartum psychosis and have used online peer support for help with this within the past two years.

**Do I have to take part?**

No. It’s completely up to you to decide whether or not you take part. Information will be provided about the study and you will be given the opportunity to ask any questions that you might have. If you choose to take part, but later change your mind, you are able to do this at any time until 01/10/2024, when the findings of the research will have been written up.

**What does it involve?**

If you decide you would like to take part, you would be asked to sign a consent form. A meeting will then be arranged, either over telephone or video conference software, such as Teams or Zoom, this will be your choice. The meeting will last around one hour and you will be asked questions about your experiences of using online peer support.

**Will I be recorded?**

The interviews will be audio recorded and the audio will be used only for the purpose of analysis. The recordings will be stored securely and no one outside of the project team will have access to the recording.

**What happens to my personal information?**

Your personal information (e.g. name, email address) will be kept confidential and stored separately to the recording of the interview. Direct quotes may be used in the final research paper but all identifiable information will be removed. The data collected for this study will be stored securely and only the project team will have access to this data. The interviews will be stored as both an audio recording and transcription, these will be anonymised as far as possible.

All data stored from this research will be destroyed after 10 years, in line with Lancaster University data management policies.

There are some limits to confidentiality: if what is said in the interview makes me think that you, or someone else, is at significant risk of harm, I may have to break confidentiality and speak to a member of staff or your care team about this. If possible, I will discuss this with you should this happen.

**What will happen to the results?**

The results will be summarised and reported in a thesis and may be submitted for publication in an academic or professional journal. The results will also be fed back to relevant stakeholders, such as postpartum psychosis forums.

**Are there any risks?**

The topics discussed in this research are sensitive and could cause some distress. During the interview, you will be allowed to discuss if you are feeling distressed, and the interview can be terminated at any point should you wish to do this. Contact details for support services will be provided at the end of the interview, should you require any further support.

**Are there any benefits to taking part?**

It is hoped that the research will help to inform the daily running of forums and make them supportive places for those who require help. In addition, you will be paid £30 for your participation as a thank you for your time.

**Who has reviewed the project?**

This study has been reviewed and approved by the Faculty of Health and Medicine Research Ethics Committee at Lancaster University.

**Where can I get further information about the study if I need it?**

If you have any questions about the study, please contact the main researcher:

Katherine Williams ([k.williams8@lancaster.ac.uk](mailto:k.williams8@lancaster.ac.uk))

**Where can I make a complaint?**

If you wish to make a complaint or raise concerns about any aspect of this study and do not want to speak to the researcher or someone outside of the Doctorate in Clinical Psychology Programme, you may also contact:

Dr Laura Machin Tel: +44 (0)1524 594973

Chair of FHM REC Email: l.machin@lancaster.ac.uk

Faculty of Health and Medicine

(Lancaster Medical School)

Lancaster University

Lancaster

LA1 4YG

**General Data Protection Regulation (GDPR)**

For further information about how Lancaster University processes personal data for research purposes and your data rights please visit our webpage: [www.lancaster.ac.uk/research/data-protection](http://www.lancaster.ac.uk/research/data-protection).

Thank you for taking the time to read this information sheet.

**Resources in the event of distress**

Should you feel distressed either as a result of taking part, or in the future, the following resources may be of assistance.

**Samaritans** (24 hour helpline to support those in distress) – 116 123

**SHOUT** (24 hour text service for those in distress) – Text “SHOUT” to 85258

**Action on Postpartum Psychosis forum** (online forum for support with postpartum psychosis) - <https://healthunlocked.com/app-network>
