# Supplementary material for: Experiences of Using Online Peer Forums Among People With Postpartum Psychosis: Interpretative Phenomenological Study
Source: JMIR Hum Factors. 2025 Dec 24;12:e80717. doi: 10.2196/80717 (PMC12780708; doi:10.2196/80717)
Supplement: Multimedia Appendix 2 [file humanfactors_v12i1e80717_app2.docx]

# **
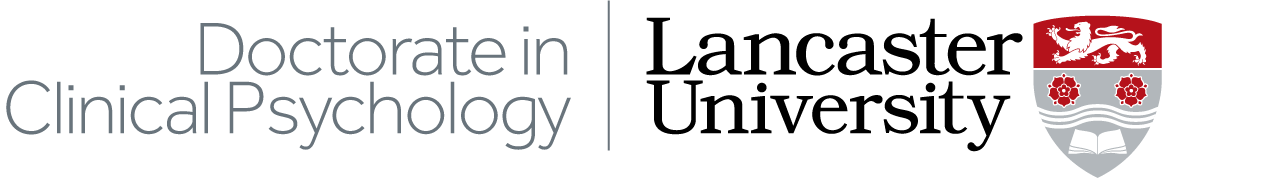
**

**Consent form**

Research title: Experiences of using online peer support for postpartum psychosis

Researcher: Katherine Williams (Trainee Clinical Psychologist)

Email contact: [k.williams8@lancaster.ac.uk](mailto:k.williams8@lancaster.ac.uk)

**Please tick each box in the table below if you consent**

| Statement | Tick box |
| --- | --- |
| 1. I confirm that I have read and understand the information sheet for the above study. I have had the opportunity to consider the information, ask questions and have had these answered satisfactorily. | 🞎 |
| 1. I understand that my participation is voluntary and that I am free to withdraw at any time, up until 01/10/2024 after which the data will have been fully integrated into the research report. I am free to withdraw without giving any reason and there will be no negative consequences of my withdrawal. | 🞎 |
| 1. I have been provided with contact details for the researcher carrying out this study. I am able to use these to withdraw from the study should I wish. | 🞎 |
| 1. I understand that any information given by me may be used in future reports, academic articles, publications or presentations by the researcher(s), but my personal information will not be included and all reasonable steps will be taken to protect the anonymity of the participants involved in this project. | 🞎 |
| 1. I agree to provide the details of a professional involved in my care (e.g. care co-ordinator, GP) in case any risk concerns need escalating. | 🞎 |
| 1. I understand that if I make any disclosures relating to risk to myself or others then this information may need to be shared with other agencies. | 🞎 |
| 1. I understand that my name will not appear in any reports, articles or presentation without my consent. | 🞎 |
| 1. I understand that any interviews will be audio-recorded and transcribed and that data will be protected and securely stored. | 🞎 |
| 1. I understand that data will be kept according to University guidelines for 10 years after the end of the study. | 🞎 |
| 1. I agree to take part in the above study. | 🞎 |

## **Participant’s details**

Participant’s name________________________________________________

Participant’s Signature____________________________________________

Date_______________

## **Declaration of researcher/person taking the consent**

I confirm that the participant was given an opportunity to ask questions about the study, and all the questions asked by the participant have been answered correctly and to the best of my ability. I confirm that the individual has not been coerced into giving consent, and the consent has been given freely and voluntarily.

Name of Researcher/person taking the consent ________________________________________

Signature of Researcher/person taking the consent ____________________________________

Date (Day/month/year) ______________

**One copy of this form will be given to the participant, and the original kept in the files of the researcher at Lancaster University**.
