# Supplementary material for: Experiences of Using Online Peer Forums Among People With Postpartum Psychosis: Interpretative Phenomenological Study
Source: JMIR Hum Factors. 2025 Dec 24;12:e80717. doi: 10.2196/80717 (PMC12780708; doi:10.2196/80717)
Supplement: Multimedia Appendix 3 [file humanfactors_v12i1e80717_app3.docx]

**Interview Schedule**

1. Can you tell me about what lead to you first deciding to use online forums for support with postpartum psychosis? *(Follow up: how did you find out about the forum?)*
2. How did you use the forum?
3. What did you value most about the forum?
4. What did you find most difficult about using the forum?
5. Can you reflect on the time in which you have used the forum and the impact this has had for you? *(Follow up: on your health and wellbeing*)
6. What changes have there been in how you feel due to using the forum?
7. What has been the most stand out moment for you when using the forum?
8. Is there anything else you would like to add that has not already been covered?
