# Supplementary material for: Experiences of Using Online Peer Forums Among People With Postpartum Psychosis: Interpretative Phenomenological Study
Source: JMIR Hum Factors. 2025 Dec 24;12:e80717. doi: 10.2196/80717 (PMC12780708; doi:10.2196/80717)
Supplement: Multimedia Appendix 4 [file humanfactors_v12i1e80717_app4.docx]

The example below provides an illustration of the double hermeneutics process and how the lead researcher made sense of the experiences discussed within interviews.

*“[Referring to more representation on forums] I feel like that’s what happening with my psychiatrist, she’s a white lady, very upper-class sort of lady and there’s a massive divide between me and her”* [Rosalind]

**Participant’s meaning**

Rosalind describes how she felt there was a divide between her and the psychiatrist that she was seeing within the community due to the differences in their demographics and backgrounds.

**Researcher’s interpretation**

The differences between Rosalind and the psychiatrist (who is often in a position of power) were felt within this relationship. The divide between them could have led to feelings of isolation and might have mirrored experiences that Rosalind has previously had as a Black African woman living within the United Kingdom.

**Reflexivity**

My own experiences of the power dynamic between psychiatrist and service user within NHS services may have led me to focus on this aspect of Rosalind’s experience. Additionally, my experience of working with those from the global majority within my clinical work led me to wonder whether this divide had previously been felt elsewhere. Having supported those who have felt silenced through this divide, I wondered whether this might have impacted Rosalind’s ability to engage on the forum freely.
