# Supplementary material for: Experiences of Using Online Peer Forums Among People With Postpartum Psychosis: Interpretative Phenomenological Study
Source: JMIR Hum Factors. 2025 Dec 24;12:e80717. doi: 10.2196/80717 (PMC12780708; doi:10.2196/80717)
Supplement: Multimedia Appendix 5 [file humanfactors_v12i1e80717_app5.docx]

| **Feeling heard, understood and supported without judgement** | | |
| --- | --- | --- |
| Interpretative Summary | Notations | Quotes |
| This theme relates to the positive feelings that the forum have contributed to for Ada. She notes that she uses the forum to relate to others, look for normalisation, sound how she is feeling and to listen to others. The impact that this on her is to feel that her mood is boosted, to feel less alone and listened to. Ada notes the positive impacts that this has on her wellbeing, such as increased confidence and self-esteem. Ada feels that peers can provide hope for the future which in turn makes her feel more positive and also makes her want to give back to the community and provide support to others. There is a feeling of shared understanding through having gone through similar experiences, even if these have not been the same | The forum boosted her mood and helped her to feel less alone through speaking to others who had gone through PP  She feels listened to and not alone on the forum  Enjoys looking at others’ posts and replies from peer support workers  Used the forum to check “is anyone else thinking this” and as a sounding board so that she didn’t burden family  Used the forum to listen to others and reach out  Wants to be a supporter, to help other women and give something back  Forum is a way to escape, open up, voice opinions and hear others’  Did open up a few times on the forum and got lots of replies which boosted her confidence  Was in a dark place but replies boosted positivity, confidence and self-esteem  It’s a nice feeling when others reply to your posts  Good to know you aren’t alone and there are women who have been through it and recovered, gives her hope | “it just helped me boost my mood, my feeling of not being alone… it very much helped me in my road to recovery actually speaking other women that are out there that have gone through similar experiences” 1.11  “really made you feel like you were listened to… you just don’t feel alone” 1.24  “I like to just look online, look at what other people are writing and saying…and just seeing the feedback that other PSWs are providing” 4.3  “I didn’t want to upset the family… so I needed the forum as my safe sounding board to go out there and just offload” 5.7  “To listen to other people’s stories and then also reach out to them” 5.11  “I was wanting to also help other women who also reach out on the forum. I always felt that I wanted to give something back” 1.28  “it was a nice way to escape into another platform where I can open up and voice my opinions, and also hear what other people were discussing at the time” 5.17  “I did open up a couple of times… and it was such a lovely feeling… I had a lot of responses… it boosts your confidence” 5.12  “I was in a dark place at the time but… it did boost my positivity, my confidence, it sort of boosted my self-esteem” 6.8  “it was a nice feeling that those people took their out just to respond to me” 6.13    “it’s always a good feeling knowing that you’re not on your own and there’s women on there that have gone through it and have recovered because that’s they thing is at the time you feel like you’re not going to get better” 14.11 |
