# Supplementary material for: Experiences of Using Online Peer Forums Among People With Postpartum Psychosis: Interpretative Phenomenological Study
Source: JMIR Hum Factors. 2025 Dec 24;12:e80717. doi: 10.2196/80717 (PMC12780708; doi:10.2196/80717)
Supplement: Multimedia Appendix 6 [file humanfactors_v12i1e80717_app6.docx]

| Theme 2 | Complementing not replacing: filling the gaps in support |
| --- | --- |
| Description | A common topic was how peer support is different to any other support that can be offered, as the outside world doesn’t understand PP causing stigma and judgement that is not felt when speaking with PEPP. Even for participants who felt that they had a support system around them throughout the diagnosis, there was discussion around how it wasn’t the same and how it felt safer to speak to peers.  However, participants also referred to services provided needed support for PP, and how actually the forums should complement this support rather than replace it entirely. Participants noted how sometimes they worry for others on the forum that they feel need professional support, as they are unsure whether the forum is being used as a replacement for this. Professional support was noted to be particularly helpful towards the start of diagnosis, due to needing information, medication and interventions in order to start recovering. However, a topic that also came up is how professional support will end at some point, feeling like “a carpet from underneath you”, and therefore the forum fills this gap, with still feeling that there is specialist support around for as long as it is needed.  Although most participants found the professional support that they received helpful, Rosalind commented on how she felt let down by services, stating that she was not understood and that systems are so overstretched that they cannot provide the support they used to be able to, and this is where peer support is having to try and fill gaps. |
| Participants contributing | Jane, Florence, Emmeline, Frida, Ada, Rosalind, Eleanor |
| Jane | *“being discharged back to the care of my GP… it felt like a carpet from underneath you… it was really frightening to have like gaps or withdrawal of things”*  *“Very grateful to have had this community… it’s something that is accessible at your own pace, like it is your choice whether to access the forum or not”*  *“I feel a bit safer to reach out in this space” [due to shared experience]*  *“as much as I feel comfortable to speak to other about in my support system… it’s not the same”*  *“but people forget what’s happened but like no one on that forum is forgetting what has happened”*  *“I’ve always been so lucky to have support… but for this experience it’s not been good enough”* |
| Florence | *“the online forum could be useful although I actually need a qualified therapist as well”*  *“some of the women sounded like they just really needed to go and see their doctor and that this forum wasn’t necessarily quite the right place for them”* |
| Emmeline | *“there wasn’t support because he didn’t have any idea what was happening and I couldn’t really explain to him… I did get some help with the kids but emotionally I had no support other than my therapists. Not because anybody didn’t want to support, they just didn’t understand at all”* |
| Frida | *“There’s such a massive need for that relation of PP because… you’re very unlikely to ever meet somebody else that’s had it… so just giving that platform is important”*  *“the outside world don’t understand it, there’s so much stigma and so much judgement from people who haven’t experienced something like this”*  *“you don’t get this kind of support from anybody who is not in the community”*  *“it’s such a different thing and it’s so, so poorly understood and so stigmatised… even people who work in like perinatal mental health services don’t thoroughly understand it”* |
| Ada | *“Peers… they’re another level because they’ve experienced it, they’ve been through it as well so you feel like you can have similar experiences”*  *“Before I knew it was like weekly… the once I get discharge from that I’m like, “right I’ve still got my platform, I’ve still got the online stuff that I can still reach out to should I need it”… it’s like you feel safe that… you’re not left to it”*  *“You’ve got the options that are available to you, so having the online support might be all that you need, but* *the health professionals, you need them at the very start of your recovery”*  *“you don’t just get left alone after the recovery, you’re always in touch with somebody”* |
| Rosalind | *“they just don’t reply and they just don’t want to speak to you because they think you’re a lunatic or crazy person. I think that having [name] there as someone who doesn’t judge me or treat me like I’m a lunatic helps because even the psychiatrist treats me like there’s something wrong with me”*  *“[About a clinical psychologist] she did actually say to me that she couldn’t relate to what I was saying and so that relationship broke down and I stopped seeing her”*  *“I think it’s the fact that people give you the time out of their day. When I’m with the NHS I feel like they’re looking at their watch… I only get to see her once every 6 months now”*  *“I still feel like no one understands. That’s why I go to the forums”*  *“It fills in where services have let me down…They said to me that care coordinators are a thing of the past now… so there’s no point to even check up on during the day or evening to say “are you alright?”*  *“It’s ridiculous that we’re having to use peer support in order to provide services that really the NHS should be providing”*  *“The NHS is saying it can’t do that, it can’t do what it used to do”*  *“I should be speaking to the professionals who understand maternal mental health, and so at the moment because I’m not getting that I’m having to speak to the ladies online”*  *“It’s the walking wounded trying to help each other”* |
| Eleanor | *“And then when you speak to other people that it’s happened to that’s the identification part of it where you’re like it wasn’t just me because you don’t quite believe it when your psychiatrist is saying to you ‘oh yeah we’ve seen your sort loads’… and that final piece of the puzzle I think for me was obviously digesting that”*  *“It’s the fact that it’s people that aren’t too close to you actually in itself can be quite therapeutic and* *you don’t want to burden your family either so talking to your people on an online forum… is I think personally the better way”* |
