# Supplementary material for: Experiences of Using Online Peer Forums Among People With Postpartum Psychosis: Interpretative Phenomenological Study
Source: JMIR Hum Factors. 2025 Dec 24;12:e80717. doi: 10.2196/80717 (PMC12780708; doi:10.2196/80717)
Supplement: Multimedia Appendix 7 [file humanfactors_v12i1e80717_app7.docx]

Table of summary of experiences of personal support and forum support

| **Personal Support (friends, family, and professionals)** | | **Forum support** | |
| --- | --- | --- | --- |
| **Benefits** | **Drawbacks** | **Benefits** | **Drawbacks** |
| They are needed in the initial stages  Provide professional help that forums cannot  Able to provide medication and interventions | Feelings of isolation  Feelings of stigma  Misunderstood  Judged  Unsafe  Feeling a burden  No shared experience  Professional services are overstretched  Potentially only short-term support  Discharge from services | Validation  Connection  Shared understanding  Growth  Increased knowledge  Sense of purpose  Provide hope  Affirmation  Reduction in loneliness  Accessible  Anonymous  Acceptance  Helping others  Safety  Long-term  Access to information | Limits of support (eg, medication and intervention)  Difficult to engage when acutely unwell  Lack of privacy  Lack of representation and diversity  Responsibility to respond  Comparison to others  Requires putting personal boundaries in place |
